# Sequential Infusions of Black Tea: Multi-Element Exposure Trade-Offs of Discarding the First Brew

**DOI:** 10.3390/foods15203613

**Published:** 2026-10-11

**Authors:** Rüveyda Esra Özkalaycı, Fatih Özkalaycı

**Affiliations:** 1 Department of Nutrition and Dietetics, Faculty of Health Sciences, Recep Tayyip Erdoğan University, 53100 Rize, Türkiye; 2 Central Research Laboratory, Karadeniz Technical University, 61080 Trabzon, Türkiye; fatihozkalayci@ktu.edu.tr

**Keywords:** black tea, sequential infusion, arsenic, lead, hazard index, trade-off, sensitivity analysis, secondary analysis, open data

## Abstract

Sample-level data preserving dependence among sequential tea infusions remain scarce. This secondary analysis examined a CC BY 4.0 dataset containing 34 black teas with three linked sequential infusions per sample. We reconstructed the cumulative element mass after consuming all three infusions versus discarding the first. The primary hazard index (HI) excluded Pb because no health-protective threshold can be established; Pb exposure and a legacy Pb-based HI were reported separately. At 10 g dry tea/day and 65 kg body weight, the median Pb-excluded HI decreased from 0.419 (IQR 0.258–0.497) to 0.260 (0.157–0.326), with a mean relative reduction of 37.1% (95% bootstrap CI 34.4–39.8%). No sample exceeded HI = 1, but this does not establish safety. When beverage volume was held at 80 mL, the mean HI reduction was only 5.7% (1.5–9.7%), showing that much of the apparent benefit reflected lower consumed volume. The total-As-based As HQ remained below one. Total-As cancer screening decreased by 35.9%; however, the proportion above 10^−4^ ranged from 0% to 64.7% across assumed inorganic-As fractions of 10–100%, demonstrating strong speciation dependence. Discarding the first infusion also reduced the estimated brewed yields of Cu, Se, and Zn by amounts equivalent to 2.4% of the adult Cu RDA, 2.5% of the Se RDA, and 0.4–0.5% of the Zn RDA; these estimates describe elemental yield in the beverage rather than absorbed nutrient intake. Overall, discarding the first infusion reduces estimated hazard indices primarily through decreased beverage volume rather than selective removal of toxic elements. Because extraction kinetics heavily depend on preparation techniques—such as traditional two-pot brewing—and regional tea chemistry, these findings cannot be directly extrapolated to Turkish teas. Instead, this study establishes a standardized exposure framework that highlights a critical knowledge gap and underscores the need for paired empirical studies evaluating local cultivars under authentic preparation practices.

## 1. Introduction

Tea (*Camellia sinensis*) is consumed daily by a large proportion of the world’s population. High consumption makes tea a relevant route for chronic exposure even when an element occurs at a low concentration. The public-health question is consequently not limited to whether dry leaves comply with a concentration limit; it also depends on extraction into the beverage, the amount of dry tea used, the number of infusions consumed, body weight, and the toxicological value used for screening [1,2,3]. High black-tea consumption and a distinctive two-pot preparation in Türkiye motivated the later methodological contextualization [4,5], but Türkiye is neither the source population nor the target of statistical inference in this international secondary analysis.

Tea plants take up both essential elements and potentially toxic elements from soil, fertilizers, atmospheric deposition, and processing environments. Country, cultivar, soil acidity, manufacturing, and packaging contribute to marked between-sample heterogeneity [1,2,3,6]. Cu, Se, and Zn are nutritionally essential, whereas As, Cd, and Pb are undesirable chronic exposures. Cr is particularly difficult to interpret because total chromium measurements do not identify oxidation state. Risk estimates based on the Cr(VI) oral reference dose are therefore conservative when speciation is unavailable. Total arsenic creates an analogous problem because the cancer slope factor is intended for inorganic arsenic [7,8,9].

The transition from leaf to cup is not a single fixed fraction. Element concentrations change across successive infusions, and the observations from one tea are correlated. The original study associated with the present open dataset showed the effects of country, tea type, and infusion process, and concluded that the first infusion generally contained the highest concentrations [1]. Direct evidence from a separate eight-infusion experiment also showed that trace-element concentrations did not consistently decline until the third infusion, and that cumulative exposure depended on the number of brews consumed [10]. These findings make infusion sequence and cumulative mass part of the exposure definition. The associated source publication did not, however, frame sequential brewing as a paired decision problem in which the total mass consumed from the same leaves is compared with the mass remaining after the first brew is discarded. Averaging three infusion concentrations can also understate exposure when a consumer drinks all three equal-volume brews, because cumulative mass is proportional to their sum rather than their arithmetic mean.

Turkish evidence has expanded but remains heterogeneous. Başaran et al. evaluated brewed black teas produced in Türkiye and found no material carcinogenic or non-carcinogenic concern under their scenarios [11]. Alkayın and Yıldız measured 54 Eastern Black Sea black teas and emphasized aluminium in infusions while reporting no limit exceedance for several other metals in leaves [12]. Demirdöğen and Kutlu measured Al, Mn, Fe, Cu, Zn, and Ni in Turkish teas and infusions [13]. Most recently, Hamitoğlu et al. studied eight bagged black-tea brands marketed in Türkiye: brand-specific HI values were 0.17–0.25, Pb cancer risk was below 10^−6^, and arsenic estimates were in the 10^−6^–10^−4^ range [14]. Turkish brewing experiments further show that preparation is part of the exposure matrix. Polat et al. found that infusion time changed mineral, chemical-quality, and sensory measures under the traditional method [4], while Cho et al. systematically demonstrated the influence of double-teapot conditions, including vessel material, water temperature, brewing method, and time [5]. These datasets differ in brewing ratio, time, analytical platform, measured elements, and exposure equations. Their published summaries are informative external benchmarks but cannot ethically or statistically substitute for unavailable sample-level observations.

Traditional Turkish preparation is not a sequence of short, directly consumed infusions. Black tea is brewed as a concentrate in an upper pot while hot water is maintained in a lower pot; the concentrate is then diluted in the glass to the drinker’s preferred strength. This system differs fundamentally from the source dataset’s three successive 5-min infusions in leaf-to-water ratio, infusion duration, thermal profile, repeated-infusion structure, vessel conditions, and final dilution. Experimental Turkish studies have examined infusion time and mineral/sensory outcomes [4] and, more recently, vessel material, water temperature, addition order, and dilution in double teapots [5]. These studies provide protocol context, but they do not supply a nationally representative, sample-level, repeated-infusion dataset. Accordingly, the present serial-infusion measurements cannot be converted into exposure estimates for the Turkish double-teapot system.

This study asked four questions. First, what fraction of the cumulative three-infusion element yield is removed by discarding the first infusion? Second, how does this paired behavior change non-carcinogenic and arsenic-related cancer screening estimates for a high-consumption adult scenario? Third, how much of the apparent change is attributable to drinking 80 rather than 120 mL, and how robust are absolute estimates of intake, body weight, Pb treatment, and assumed inorganic-As fraction? Fourth, is there evidence of between-country heterogeneity in this limited sample, and what reduction in the brewed yields of Cu, Se, and Zn does this entail relative to adult dietary reference intakes? The analysis implements a protocol-agnostic, standardized secondary-analysis workflow for linked sequential infusions. Relative to the source publication, it adds cumulative mass balance, paired strategy effects with uncertainty, equal-volume and toxicological sensitivity analyses, nutritional-reference comparisons, zero-value diagnostics, and study-level Turkish contextualization. It does not claim that the international samples represent Turkish tea production.

## 2. Materials and Methods

### 2.1. Study Design and Data Provenance

This was a secondary analysis of non-personal laboratory measurements. The sample-level dataset was downloaded from Mendeley Data (Version 1; DOI: 10.17632/4md98yy6c4.1), where it is distributed under CC BY 4.0 [15]. The associated peer-reviewed study analyzed 45 commercial teas from China, India, and the United States for Ag, As, Ba, Cd, Cr, Cu, Pb, Se, and Zn in dry leaves and three infusions [1]. The present analysis used the infusion measurements and preserved the unique sample identifier linking the three successive infusions from each tea.

The laboratory protocol was inherited from the source study rather than performed by the present authors. According to the source publication, 15 marketed teas were obtained from each country. For each infusion, 1.5 g tea was combined with 40 mL boiled deionized water, gently shaken for 5 min, and filtered through Whatman No. 1 paper, and the same leaves were infused twice more. Leaf digests and infusion samples were prepared in duplicate because the available leaf mass was limited, and nine elements were determined using an inductively coupled plasma optical emission spectrometry (ICP-OES, 5100 Agilent Technologies, Santa Clara, CA, USA) [1]. Replicate-level readings, sample-specific detection limits, recoveries, and measurement-uncertainty components were not separately identified in the deposited worksheet; therefore, laboratory repeatability could not be independently re-estimated in this secondary analysis.

The dataset contained 34 black teas (China *n* = 10, India *n* = 15, United States *n* = 9) and 11 green teas. Because black tea dominates consumption in Türkiye, the black-tea subset was specified as the primary analytic population. All 45 teas formed a sensitivity population. No row was generated from a mean, standard deviation, or range. Published Turkish values were retained as study-level summaries and were not appended to the sample-level matrix. Table 1 summarizes data provenance, analytic level, and permitted use.

### 2.2. Exposure Reconstruction for Sequential Infusions

The source experiment used 1.5 g dry tea and 0.04 L infusion volume. For element *i*, sample *j*, and infusion *s*, the measured concentration *C_ijs_* (micrograms/L) was converted to metal content normalized to dry tea, *M_ijs_* (mg/kg dry tea), as follows:

*M_ijs_* = *C_ijs_* × *V*/*W*

where *V* is 0.04 L and *W* is 1.5 g. Because micrograms per gram and milligrams per kilogram are numerically equivalent, no additional scale factor is required. Cumulative content after drinking all three infusions was *M_ij_*(*all*) = *M*_*ij*1_ + *M*_*ij*2_ + *M*_*ij*3_. Content after discarding the first infusion was *M_ij_*(discard) = *M*_*ij*2_ + *M*_*ij*3_. This mass-balance definition differs from averaging concentrations and is appropriate when all equal-volume sequential brews are consumed.

The primary high-consumption scenario retained the source workbook assumptions of 10 g dry tea/day and 65 kg body weight. Sensitivity scenarios crossed intakes of 5, 10, and 15 g/day with body weights of 65, 70, 75, 80, and 85 kg, yielding 15 intake-body-weight combinations for each strategy. Because the all-infusion strategy represented 120 mL and the discard-first strategy 80 mL per 1.5 g leaves, an equal-volume sensitivity comparison scaled the pooled three-infusion mass to 80 mL (two-thirds of the 120 mL total) and compared it with the observed second-plus-third mass. This normalization estimates whether removing the first infusion changes exposure beyond the one-third reduction in beverage volume; it assumes that the combined 120 mL can be represented by its volume-weighted mean concentration.

The average daily dose (ADD, mg/kg body weight/day) was calculated as:

ADD*_ij_* = (*M_ij_* × IR)/(1000 × BW)

where IR is dry-tea intake in g/day and BW is body weight in kg. The calculation assumes daily long-term consumption. It quantifies exposure attributable to tea infusion only and does not include food, water, or other sources.

### 2.3. Risk-Screening Metrics and Toxicological Caveats

Element-specific hazard quotients were calculated as HQ*_i_* = ADD*_i_*/RfD*_i_*. The primary HI summed Ag, As, Ba, Cd, Cr, Cu, Se, and Zn, but excluded Pb. Reference doses were retained from the source workbook to enable a reproducible reanalysis: Ag 0.005, As 0.0003, Ba 0.2, Cd 0.0005, Cr 0.003, Cu 0.04, Pb 0.025/7, Se 0.005, and Zn 0.3 mg/kg/day [15,16,17]. HI < 1 was treated only as a screening comparison with selected reference values and not as evidence of safety.

Pb exposure was reported separately as ADD. For auditability only, a sensitivity HI used the source workbook’s historical JECFA PTWI proxy of 0.025/7 mg/kg/day. JECFA withdrew that PTWI because no health-protective tolerable exposure could be identified, and EFSA likewise concluded that a threshold could not be established [18,19]. The legacy Pb HQ therefore has no current safe-threshold interpretation. Total Cr was paired with the Cr (VI) RfD although species were unavailable; Cr results were retained as conservative screening values, not compound-specific risk estimates.

For carcinogenic screening, total arsenic exposure was multiplied by the source workbook slope factor of 1.5 (mg/kg/day)^−1^. Because that factor applies to inorganic As, sensitivity analyses assumed that 10%, 20%, 29%, 50%, 75%, 88%, or 100% of measured total As was inorganic. The 29–88% envelope was selected to span values reported in tea-speciation studies; 10% and 20% were added as lower-bound scenarios, while 100% retained the original worst-case screen [20,21]. These are scenario calculations rather than measured speciation. Values above 10^−4^ are reported as screening exceedances, not cancer probabilities or predicted incidence; the current EFSA assessment uses a margin-of-exposure approach for inorganic As [9]. Non-cancer arsenic screening was reported separately as a total-As-based As HQ using the selected oral RfD; because speciation was unavailable, this HQ is also an upper-bound screen rather than a measured inorganic-As estimate.

### 2.4. Statistical Analysis

Analyses were conducted at the tea-sample level. Infusion-order differences were evaluated separately for each element using a permutation-based Friedman statistic (20,000 within-sample permutations); Kendall’s W was reported as the effect size, and Benjamini–Hochberg control was applied across nine elements [22]. First-infusion share was defined only when the three-infusion total was greater than zero. First-share intervals used 10,000 bootstrap resamples; strategy-effect intervals used 20,000 [23]. Zeros were retained exactly as reported because sample-specific limits of detection and the source preprocessing status of zeros were unavailable. We report zero counts, element-specific effective sample sizes, and a complete-positive sensitivity estimate; substitution-based censored-data analyses were not attempted because they would require unknown detection limits.

Risk outcomes were summarized by medians, interquartile ranges, means, absolute paired reductions, relative paired reductions, and sample-level bootstrap 95% confidence intervals. Because the discard-first outcome is algebraically nested within the all-infusion outcome, no hypothesis test was used to present the reduction as an independent intervention effect. Between-country heterogeneity was treated as exploratory and was tested by permutation of country labels (50,000 permutations). Fixed seeds were used for all resampling. To evaluate whether the 34-sample result was driven by one influential tea, the mean relative reductions were recalculated after omitting each sample in turn. A complementary leave-one-country-out analysis assessed dependence on any single country group. These diagnostics assess internal stability, not population representativeness or laboratory repeatability. The data analyses were performed using Python 3.12 (Python Software Foundation, Wilmington, DE, USA).

### 2.5. External Contextualization with Turkish Studies

External contextualization used a targeted, study-level comparison rather than a systematic review or meta-analysis. We extracted only published sample counts, element panels, infusion protocols, concentration ranges, and risk summaries from the four Turkish reports available for this purpose [11,12,13,14]. Values were harmonized conceptually by element and matrix; no published mean or range was expanded into pseudo-observations. Direct numerical comparison was limited to clearly comparable units, and protocol differences were carried into the interpretation. The small and heterogeneous evidence base provides contextual plausibility checks only, not external validation. Separately, two Turkish brewing-protocol experiments [4,5] were used only to characterize the traditional double-teapot system and to define the protocol gap requiring future experimental validation; they were not treated as exposure or risk-validation datasets.

### 2.6. Standardized Analytical Workflow

**Figure 1:**
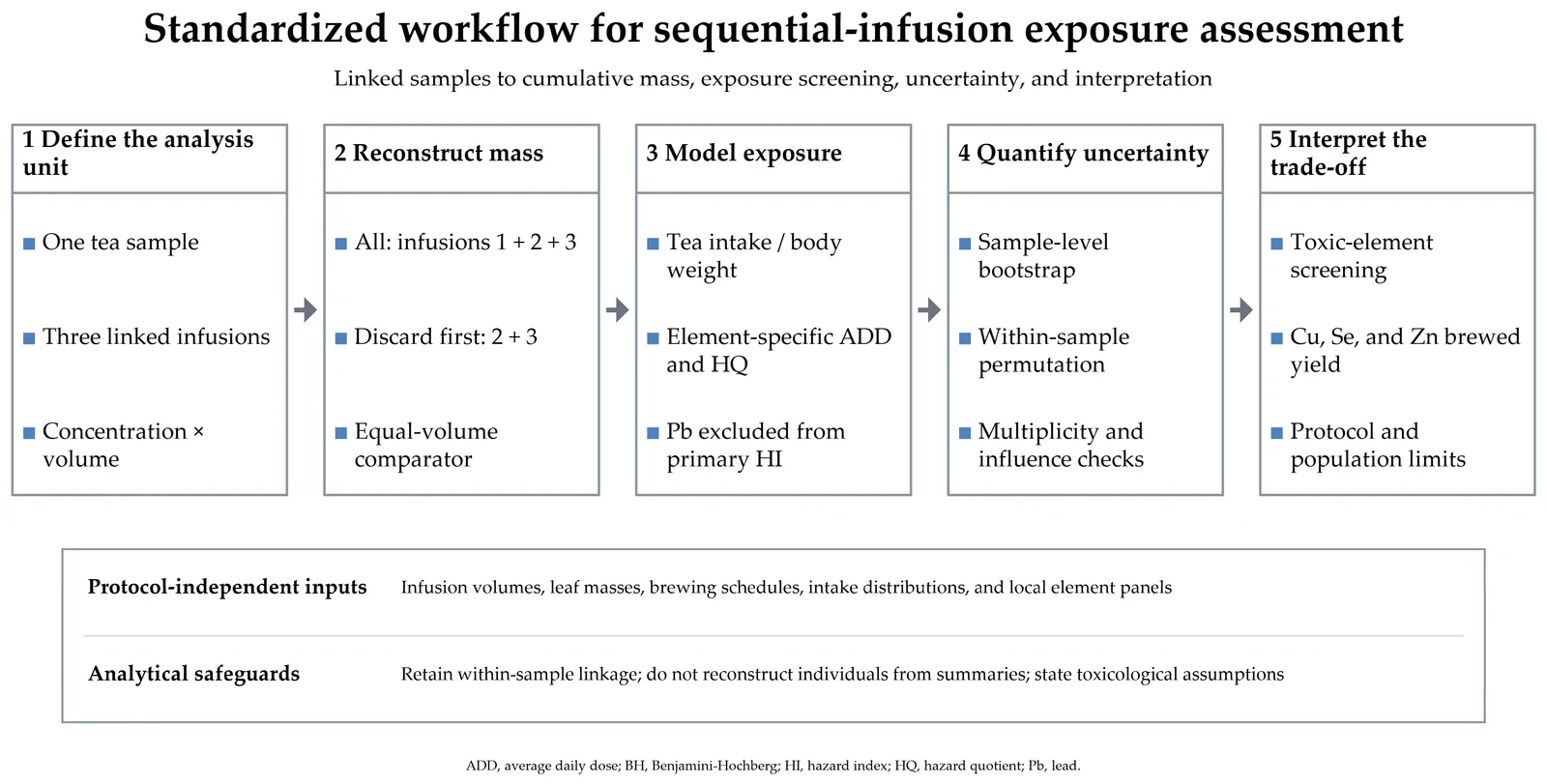
Standardized workflow for sequential-infusion exposure assessment. ADD, average daily dose; BH, Benjamini–Hochberg; HI, hazard index; HQ, hazard quotient; Pb, lead. The diagram distinguishes measured inputs, linked cumulative-mass reconstruction, exposure modelling, uncertainty analysis, and constrained interpretation.

The workflow is protocol-agnostic in the sense that the linked-sample equations accept alternative infusion volumes, leaf masses, brewing schedules, intake distributions, and locally measured element panels. Its required guardrails are preservation of within-tea linkage, reconstruction of cumulative consumed mass, explicit toxicological assumptions, sample-level uncertainty analysis, and refusal to expand published aggregate summaries into pseudo-observations (Figure 1). This is a standardized secondary-analysis workflow, not a new laboratory platform or a meta-analysis.

## 3. Results

### 3.1. Serial Infusion Profiles

**Figure 2:**
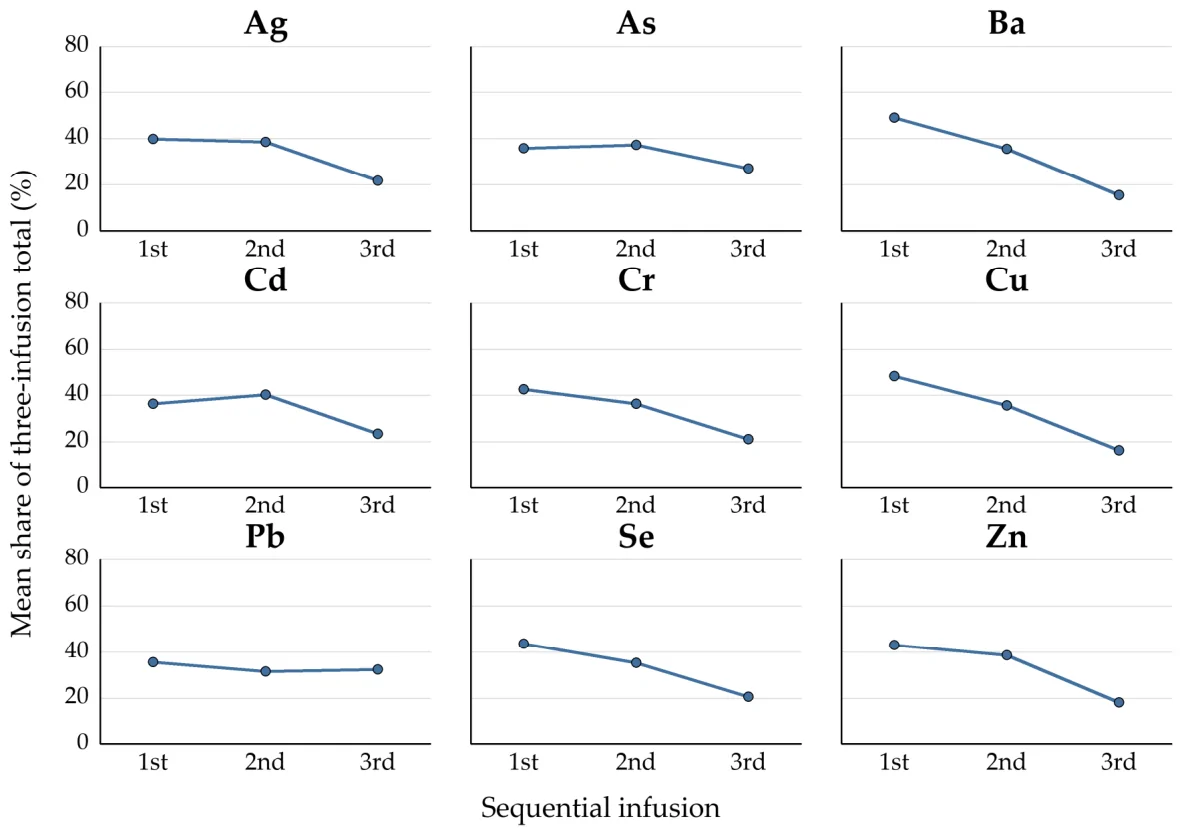
Mean share of each element’s cumulative three-infusion concentration by sequential infusion in black tea (*n* = 34). Supplementary Figure S1 provides sample-level distributions; Table 2 reports inferential statistics and effective sample sizes. Points are element-specific mean shares connected across infusion order; lines guide interpretation of the sequential pattern and do not represent fitted trajectories. Error bars were omitted to avoid conflating descriptive shares with inferential uncertainty; Table 2 reports effect sizes, adjusted q values, and bootstrap intervals, and Supplementary Figure S1 shows the sample-level distributions.

In the 34 black teas, infusion order influenced Ag, As, Ba, Cr, Cu, Se, and Zn after false-discovery-rate control (all *q* <= 0.006). Cd showed weak evidence after adjustment (*q* = 0.057), and Pb did not vary detectably by infusion order (*q* = 0.418). The strongest ordering effects were observed for Cu (Kendall’s *W* = 0.805), Ba (*W* = 0.781), Cr (*W* = 0.649), and Zn (*W* = 0.592). As showed a smaller but statistically supported ordering effect (*W* = 0.151). The element-specific pattern was not uniformly monotonic: median Zn and Cr concentrations were slightly higher in the second than the first infusion, illustrating why a single global transfer factor is insufficient (Table 2; Figure 2). Detailed infusion statistics and first-infusion shares are provided in Supplementary Tables S1 and S2, sample-level distributions in Supplementary Figure S1, and zero/effective-n diagnostics in Supplementary Tables S3 and S4.

### 3.2. Exposure Reduction and Essential-Element Trade-Off

**Figure 3:**
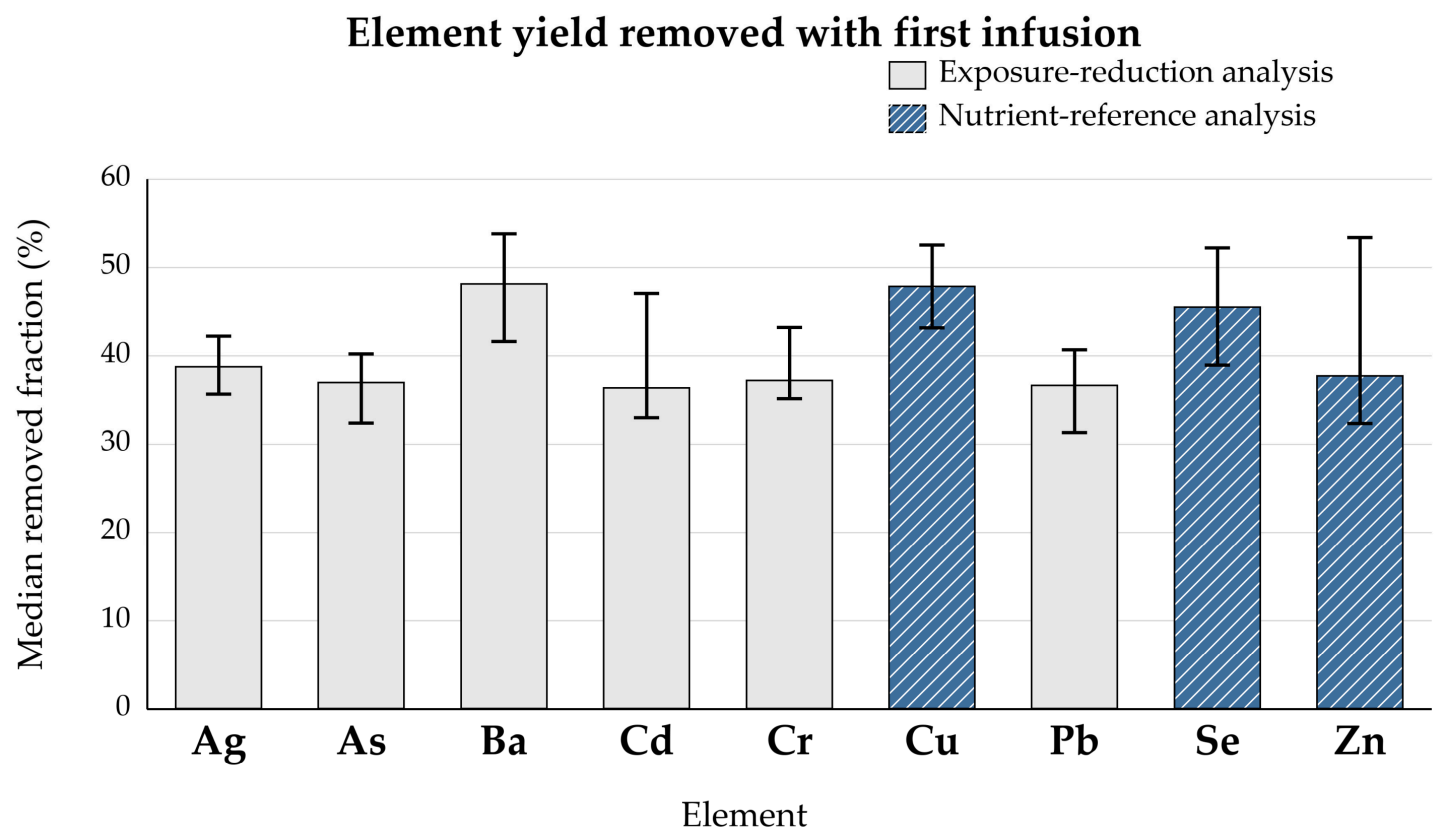
Median fraction of the cumulative three-infusion element yield removed by discarding the first infusion. Cu, Se, and Zn are highlighted as essential nutrients evaluated in the nutritional comparison; no essential/non-essential classification is assigned to the remaining elements because interpretation can depend on chemical species. Error bars are 95% sample-level bootstrap confidence intervals.

Discarding the first infusion removed median shares of 37.1% of cumulative As, 36.7% of Pb, 36.4% of Cd, and 37.3% of Cr. The estimated reductions in brewed yield were 21.5 micrograms Cu (IQR 16.9–27.7), 1.37 micrograms Se (0.59–2.19), and 43.9 micrograms Zn (31.3–76.0) in the 10 g/day scenario. These correspond to 2.4% of the adult Cu RDA, 2.5% of the Se RDA, and 0.4% (men) to 0.5% (women) of the Zn RDA [24,25,26,27,28]. These values represent elemental yield in the beverage, not absorbed or bioavailable nutrient intake. The complete calculation is reported in Supplementary Table S5 and summarized in Figure 3.

### 3.3. Paired Health-Risk Screening

The primary Pb-excluded median HI was 0.419 (IQR 0.258–0.497) for consuming all three infusions and 0.260 (0.157–0.326) after discarding the first. The mean paired absolute reduction was 0.152 (95% bootstrap CI 0.128–0.177), and the mean relative reduction was 37.1% (34.4–39.8%). No sample exceeded HI = 1 under the primary assumptions, but this finding does not establish safety. In the legacy Pb-HQ sensitivity analysis, medians were 0.434 and 0.273, the mean absolute reduction was 0.156 (0.133–0.181), and the mean relative reduction was 37.1% (34.4–39.7%), showing that removal of the obsolete Pb quotient changed the absolute HI but not the paired contrast. Median Pb ADD was 0.0297 micrograms/kg body weight/day for all infusions and 0.0179 after discarding the first. Main and legacy-Pb sensitivity results are summarized in Table 3 and Table S6. The separately reported total-As-based As HQ had medians of 0.330 (IQR 0.200–0.430) and 0.224 (0.129–0.288), respectively; no sample exceeded As HQ = 1. Because total rather than inorganic As was measured, these HQ values are upper-bound screening estimates.

**Figure 4:**
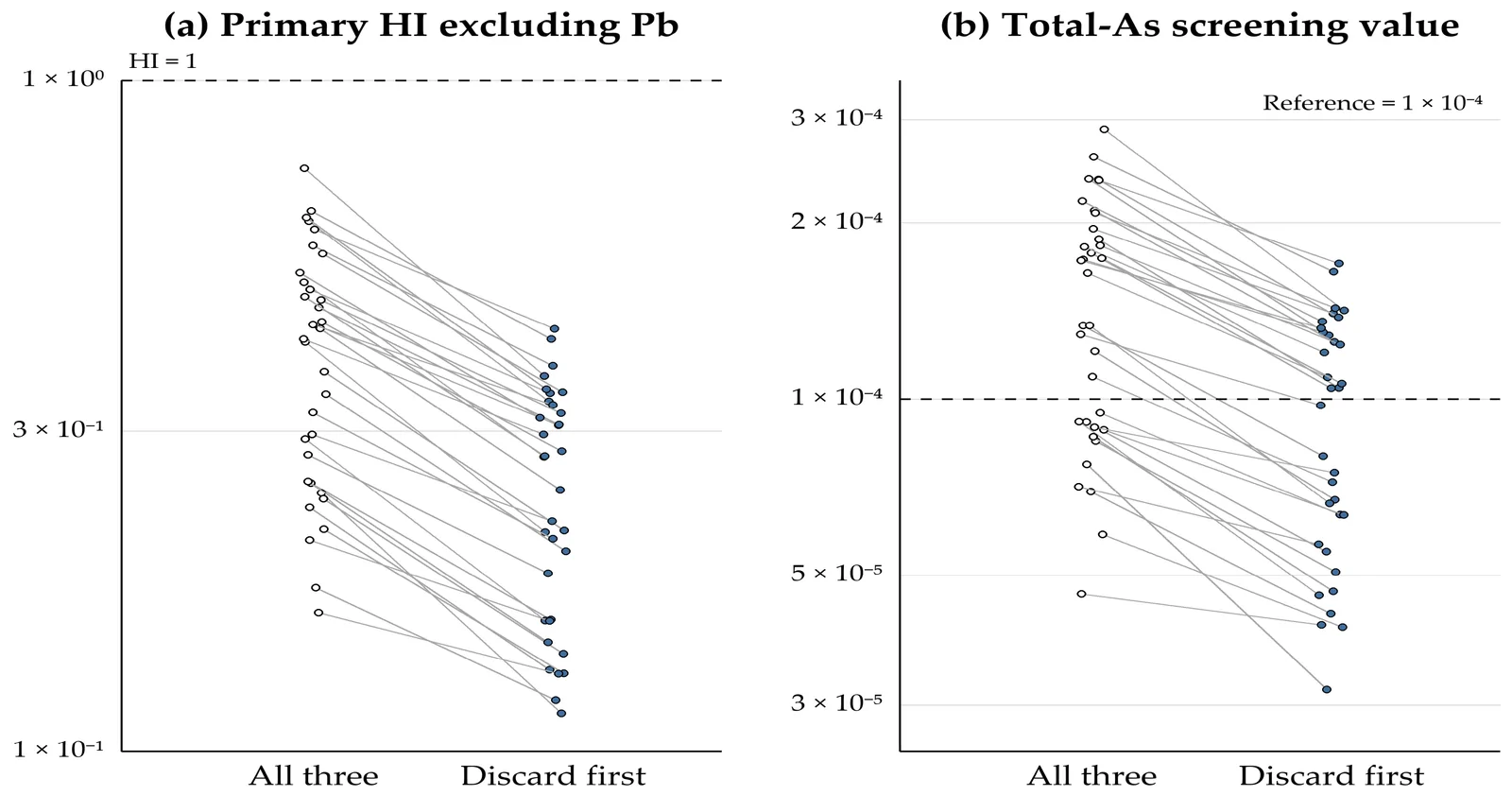
Sample-level paired change in (**a**) primary hazard index excluding Pb and (**b**) total-As screening value after discarding the first infusion. Dashed reference lines indicate HI = 1 and arsenic screening value = 10^−4^. The Pb-excluded HI is the primary result; total-As values represent the 100%-inorganic worst-case scenario.

Under the 100%-inorganic worst-case assumption, median arsenic cancer-screening values were 1.49 × 10^−4^ for all three infusions and 1.01 × 10^−4^ after discarding the first. The mean absolute reduction was 5.49 × 10^−5^ (95% bootstrap CI 4.54 × 10^−5^–6.50 × 10^−5^), and the mean relative decrease was 35.9% (32.4–39.2%). The number above 10^−4^ changed from 22/34 (64.7%) to 17/34 (50.0%). Results depended strongly on the assumed inorganic fraction: exceedances for all/discard strategies were 0/0 at 10%, 0/0 at 20%, 0/0 at 29%, 8/0 at 50%, 19/7 at 75%, 21/13 at 88%, and 22/17 at 100%. These values are upper-bound screening scenarios, not measured cancer probabilities or predicted incidence. Assumed inorganic-fraction results are reported in Supplementary Table S7 and paired sample-level changes in Figure 4.

### 3.4. Robustness Across Origins and Tea Types

No evidence of between-country heterogeneity in relative Pb-excluded HI reductions was detected in this limited sample (China *n* = 10, India *n* = 15, United States *n* = 9; permutation *p* = 0.922). This null result does not demonstrate equivalence or transportability across countries. Across the 15 intake-body-weight combinations, median Pb-excluded HI ranged from 0.160 (5 g/day, 85 kg) to 0.629 (15 g/day, 65 kg) for all infusions and from 0.099 to 0.390 after discarding the first. One sample exceeded HI = 1 in the 15 g/day all-infusion scenarios at 65 and 70 kg; no sample exceeded HI = 1 after the first infusion was discarded. Relative reductions were invariant to proportional changes in intake and body weight, whereas absolute HI, total-As-based HQ, total-As screening values, and Pb ADD scaled directly. In the equal-80-mL comparison, discarding the first infusion produced only a 5.7% mean HI reduction (95% CI 1.5–9.7%) and a 3.8% mean total-As reduction (−1.5% to 8.9%). Some samples had negative equal-volume reductions, confirming that the unadjusted 37% contrast combines a sequence-composition effect with reduced beverage volume (Supplementary Tables S8 and S9). Detailed intake/body-weight, equal-volume, all-tea, and country results are reported in Supplementary Tables S8–S11, respectively. Leave-one-sample-out means were 36.6–37.7% for primary HI reduction and 35.2–36.6% for total-As screening reduction; the maximum shifts from the full-sample means were 0.55 and 0.74 percentage points, respectively. For the equal-80-mL contrast, the corresponding ranges were 5.0–6.5% and 2.8–4.9%. Leave-one-country-out estimates ranged from 36.8% to 37.4% for primary HI; this internal diagnostic neither resolves limited geographic sampling nor supports extrapolation to tea grown in Türkiye or other unmeasured populations. Full influence results are reported in Supplementary Table S12.

### 3.5. Study-Level Turkish External Context

The normalized median As content of the first infusion in the open black-tea data was 0.232 mg/kg dry-tea equivalent, within the 0.1–0.3 micrograms/g range reported for Turkish marketed bagged-tea infusions [14]. The corresponding first-infusion medians for Pb, Cu, and Zn were 0.072, 2.15, and 4.39 mg/kg, respectively. Hamitoğlu et al. reported wider or higher study-level ranges for Pb (0.1–0.5 micrograms/g), Cu (13.5–17.8 micrograms/g), and Zn (9.4–16.9 micrograms/g), under a different 6 g/200 mL, 5 min bagged-tea protocol [14]. Because the matrix normalization and brewing procedures were not identical, agreement for As and differences for the other elements were treated as context rather than equivalence. Study-level Turkish protocols and comparability limits are summarized in Table 4. Table 4 separates exposure/risk evidence from brewing-protocol evidence so that preparation studies are not misinterpreted as external validation of the present risk estimates.

The Turkish study’s brand-specific HI values (0.17–0.25) overlapped the lower portion of the present sample-level distribution and were close to the discard-first legacy-Pb HI median (0.273), although its HI included Al, used different body-weight and lifetime parameters, and was based on one infusion [14]. Both analyses nevertheless converged on two qualitative findings: HI remained below unity and cancer screening was driven by total arsenic. Başaran et al. also recommended lighter brewing, while Alkayın and Yıldız identified Al as a regional issue requiring attention [11,12]. These observations reinforce the need for Turkish sample-level multi-infusion data that include Al and arsenic speciation.

## 4. Discussion

### 4.1. Principal Findings

This reanalysis quantifies both the composition and volume effects of feasible brewing behavior. The unadjusted strategy comparison reduced primary HI by 37.1%, but the equal-volume sensitivity estimate reduced it by only 5.7%. Accordingly, discarding the first brew mainly lowers exposure because less beverage is consumed, with a smaller and sample-dependent contribution from the elemental profile of the first infusion. The result should not be presented as proof of a toxic-element-selective intervention.

A newly published probabilistic tea-rinsing study is the closest comparator to the consumer question addressed here [29]. Hu et al. reported that rinsing reduced mean adult total carcinogenic risk by 36.2%, with arsenic as the dominant contributor. That convergence supports the direction of the paired contrast but does not validate the present absolute estimates because the tea products, rinsing protocol, exposure model, and probabilistic assumptions differed. Xiao et al.’s eight-infusion experiment provides the closest comparator for linked successive infusions [10]: the second infusion remained higher than the subsequent six for several elements, and cumulative HI reached 1.08 for Tieguanyin tea when all eight short infusions were included. In contrast, no HI exceeded one in the present three-infusion black-tea analysis. Together, these studies show that a fixed ‘first-brew removal percentage’ should not be generalized across protocols. The present contribution is the sample-linked mass-balance comparison, explicit equal-volume decomposition, arsenic-speciation scenarios, and essential-element trade-off for a defined three-infusion dataset.

The independent contribution is a protocol-agnostic, standardized analytical workflow and is decision-focused, not a claim that declining concentrations across successive infusions or tea rinsing are newly discovered. The associated source publication evaluated country, tea type, and infusion effects using the supplied workbook. Xiao et al. followed eight short infusions and established protocol-specific successive-infusion patterns [10], while Hu et al. directly evaluated tea rinsing using a probabilistic risk model [29]. The present reanalysis addresses a narrower linked-sample question: a defined discard-first contrast in black-tea samples. It reconstructs cumulative mass, reports paired absolute and relative effects with bootstrap uncertainty, separates the one-third volume reduction from sequence composition, excludes Pb from the primary threshold-based HI, varies inorganic-As fraction and intake/body weight, quantifies reductions in brewed essential-element yield, and adds zero-value and influence diagnostics. The equal-volume result materially changes interpretation: most of the unadjusted 37.1% reduction reflects consuming less beverage, not selective removal of hazardous elements. Figure 1 visualizes the workflow, and Supplementary Table S13 directly compares the source study with the present analysis. This is neither a new experimental platform nor a formal meta-analysis.

Recent risk studies also support separating analytical concentration from the exposure model. Esfarjani et al. used Monte Carlo simulation for 23 black teas and reported HQ values below 0.01, while arsenic cancer-risk estimates for foreign teas lay mainly within the 10^−6^–10^−4^ interval [30]. Ahmed et al. likewise reported HI below one and negligible cumulative cancer risk in tea infusions analyzed by ICP-OES [31]. Their broadly reassuring non-cancer results agree with the absence of HI exceedances here, but the absolute estimates are not interchangeable. Leaf mass, water volume, brewing duration, age group, element panel, and toxicological inputs differed. This protocol sensitivity explains why the present paired relative reduction is more transportable than its absolute HI value.

### 4.2. Benefit-Risk Interpretation

The measured intervention was not selective and does not constitute a complete risk–benefit assessment as defined in current EFSA guidance, which requires explicit characterization of relevant beneficial and adverse effects, exposure, variability, and uncertainty [32]. Although discarding the first infusion reduced the brewed amounts of Cu, Se, and Zn, the median reductions corresponded to only approximately 0.4–2.5% of adult RDAs. These values represent elemental yield in the beverage, not absorbed or bioavailable nutrient intake. However, polyphenols, caffeine, fluoride, sensory properties, bioaccessibility, and other bioactive constituents were not measured. The defensible description is therefore a multi-element exposure trade-off, not a net health benefit.

Recent extraction studies make the unmeasured benefit side of this trade-off more concrete. Under a Turkish black-tea protocol, Polat et al. observed transfer rates of 69.8–90.2% for chemical-quality parameters but 25.1–41.8% for minerals, with infusion time also changing sensory acceptance [4]. In a 2026 green-tea study, Zhang et al. found that successive infusions redistributed polyphenols, amino acids, caffeine, volatile compounds, and sensory attributes; the second infusion had the highest overall extraction efficiency for several major bioactives [33]. Green and black teas cannot be equated, yet both studies show that discarding an early brew is unlikely to be compositionally neutral. A consumer recommendation therefore requires co-measurement of toxic elements, essential elements, caffeine, polyphenols, bioaccessibility, and sensory acceptability in the same black-tea experiment.

As remained the dominant screening driver, but non-cancer HQ and cancer screening require separate interpretation. The total-As-based As HQ remained below one for every sample and is an upper-bound non-cancer screen because inorganic As was not measured. For cancer screening, no sample exceeded 10^−4^ at assumed inorganic fractions of 10%, 20%, or 29%, whereas counts increased to 22/34 and 17/34 at 100%. This scenario spread, rather than the worst-case value alone, is the central result. Before drawing health conclusions, direct speciation and a contemporary margin-of-exposure framework are needed.

### 4.3. Türkiye as an External Methodological Context

The Turkish literature provides protocol and magnitude context but remains too small and heterogeneous for pooled inference. Four exposure/risk reports differ in tea form, element panel, brewing ratio, duration, analytical platform, and exposure equations, while two additional experiments characterize traditional brewing conditions rather than toxic-element risk. None provides evidence that the international samples represent exposure among Turkish consumers. The contribution for Türkiye is therefore methodological: the paired mass-balance and sensitivity framework can guide a future Turkish experiment, but the present section does not estimate Turkish exposure, compare national risk, or imply that the three-country sample represents Turkish-grown tea.

The 2026 Turkish brewing experiment by Cho et al. makes the protocol gap especially clear [5]. It tested porcelain and stainless-steel double teapots, two orders of adding tea and water, temperatures of 75, 90, and 95 °C, and brewing times of 15 and 20 min; the concentrate was then diluted for serving. Those conditions differ materially from the source dataset’s 1.5 g in 40 mL, three-successive-infusion design. Because extraction is protocol-dependent, neither the concentrated upper-pot phase nor the final diluted serving can be inferred from the present serial-infusion data. This non-equivalence is the rationale for, rather than evidence from, a future experimental validation in Türkiye.

A future validation study should analyze the same Turkish black-tea lots under a prespecified, realistic double-teapot protocol and, where feasible, a matched successive-infusion comparator. It should measure both the upper-pot concentrate and the final diluted glass at predefined dilution ratios; record leaf-to-water ratio, time, temperature, vessel material, water pH, hardness, and background elements; include independent brewing replicates, water blanks, detection and quantification limits, certified-reference recoveries, and measurement uncertainty; and deposit anonymized sample-level data. The element panel should include at least As with speciation, Al, Cd, Cr with speciation where feasible, Cu, Pb, Se, and Zn. Such a paired design would test both the transferability of the analytical workflow and the actual exposure consequences of Turkish double-teapot preparation without treating study-level summaries as pseudo-observations.

### 4.4. Strengths and Limitations

Strengths include use of an openly licensed sample-level dataset, explicit preservation of within-tea dependence, cumulative mass balance, paired effect sizes with bootstrap uncertainty, equal-volume normalization, Pb and arsenic sensitivity analyses, intake/body-weight scenarios, nutrient-reference comparisons, multiplicity control for infusion profiles, and explicit zero-value diagnostics. The full computational workflow, derived tables, fixed seed, package versions, and replication instructions are supplied as Supplementary Code S1.

Limitations are substantial. The analytic sample comprised 34 black teas, was not collected in Türkiye, and is not nationally representative. Leave-one-out stability shows that no single sample drove the mean contrast, but it does not replace a larger independent sample. Although the source study reported duplicate preparation, it did not deposit replicate-level values, so laboratory repeatability and measurement uncertainty could not be independently quantified. The source brewing ratio differs from common two-pot preparation. Exposure scenarios omit food, water, and other beverages. Elemental species were unavailable. The primary HI excludes Pb because a valid threshold is unavailable, while the legacy Pb quotient is shown only for comparison; total Cr still uses a Cr (VI) reference value. Arsenic-fraction analyses are hypothetical, not measured. Zeros could not be classified as true zeros or non-detects, and censored-data substitution was impossible without sample-specific detection limits. Finally, the equal-volume analysis assumes a volume-weighted pooled comparator and does not replace a randomized brewing experiment. Background dietary intake of Cu, Se, and Zn was unavailable, so the RDA comparison describes the reduction in tea-derived brewed yield but not its effect on individual nutrient adequacy. Water chemistry was also unavailable and may limit transfer of the extraction profile across regions.

The extraction uncertainty is supported by recent experimental evidence. Długaszek and Mierczyk found that both brewing time and pH changed element release from green tea, with particularly marked effects for Al, Ca, and Mn [34]. Wu et al. likewise reported element-specific effects of time and temperature on toxic-element leaching from oolong tea [35]. Although both tea types differ from black tea, these studies identify water chemistry and thermal conditions as plausible sources of between-study heterogeneity. Future black-tea work should record water pH, hardness, and background element concentrations, analyze water blanks, and report the composition of the brewing vessel alongside time, temperature, leaf-to-water ratio, and dilution.

## 5. Conclusions

In this internally stable but geographically limited sample of 34 black teas, discarding the first of three 40-mL infusions reduced the primary Pb-excluded HI by a mean 37.1%, but the reduction was only 5.7% when beverage volume was held at 80 mL. Thus, most of the unadjusted decrease reflected lower consumed volume rather than selective removal of hazardous elements. Furthermore, total-As screening results varied markedly with the assumed inorganic fraction, and Pb could not be interpreted with a threshold-based HQ. While the estimated reductions in brewed yield of Cu, Se, and Zn corresponded to only approximately 0.4–2.5% of adult RDAs, these values represent elemental yield in the beverage rather than absorbed or bioavailable nutrient intake; the unquantified bioactive profile further limits a full risk–benefit assessment. These findings support establishing a reproducible multi-element exposure framework rather than a universal recommendation to discard the first brew. Because elemental extraction is sensitive to preparation conditions—such as traditional two-pot brewing—and regional tea chemistry, these estimates cannot be extrapolated to tea produced or consumed in Türkiye. This methodological boundary identifies an important empirical gap in the current toxicological literature. Consequently, this study provides the theoretical and analytical rationale for a future paired experimental validation in Türkiye, incorporating Turkish sample-level data, authentic two-pot extraction dynamics, local water chemistry, and elemental speciation.

## Figures and Tables

**Table 1:** Data sources, analytic level, and permitted role in the reanalysis.

Source	Material	Analytic Level	Role
Na Nagara et al. [1,15]	45 teas; 3 infusions; 9 elements	Sample-level, repeated	Primary reanalysis; CC BY 4.0
Hamitoğlu et al. [14]	8 bagged black-tea brands in Türkiye	Published brand summaries	External benchmark only
Başaran et al. [11]	Brewed black teas produced in Türkiye	Published summaries	Qualitative risk context
Alkayın and Yıldız [12]	54 Eastern Black Sea black teas	Published summaries	Regional context; Al emphasis
Demirdöğen and Kutlu [13]	Turkish teas and infusions	Published summaries	Protocol and element context

Turkish published summaries were not treated as raw observations and were not pooled in individual-level regression.

**Table 2:** Sequential infusion profile and fraction removed by discarding the first infusion in black tea (*n* = 34).

Element	First Median	Second Median	Third Median	First Share, % (95% CI)	Kendall *W*	BH *q*
Ag	0.403	0.34	0.158	38.8 (35.7–42.3); *n* = 34	0.343	0.0001
As	8.69	8.5	6.38	37.1 (32.4–40.3); *n* = 34	0.151	0.0058
Ba	27.4	26.2	11.6	48.2 (41.6–53.8); *n* = 34	0.781	0.0001
Cd	0	0	0	36.4 (33.0–47.1); *n* = 16	0.087	0.0569
Cr	6.26	6.64	4.08	37.3 (35.2–43.2); *n* = 34	0.649	0.0001
Cu	80.6	65.3	28.6	48.0 (43.2–52.6); *n* = 34	0.805	0.0001
Pb	2.7	2.66	2.4	36.7 (31.4–40.7); *n* = 34	0.027	0.4176
Se	5.13	3.72	1.98	45.6 (39.0–52.2); *n* = 28	0.282	0.0001
Zn	165	186	80.3	37.8 (32.4–53.4); *n* = 34	0.592	0.0001

Concentrations are micrograms/L. First-infusion shares are medians with sample-level bootstrap 95% CIs; n is the element-specific number of teas with a positive three-infusion total. Effective *n* was 16 for Cd, 28 for Se, and 34 for all other elements.

**Table 3:** Paired risk-screening outcomes for black tea under sequential-infusion strategies (*n* = 34).

Outcome	All Three Infusions	Discard First	Mean Paired Reduction (95% CI), Absolute; Relative
Primary HI (Pb excluded)	0.419 (0.258–0.497)	0.260 (0.157–0.326)	0.152 (0.128–0.177); 37.1% (34.4–39.8)
Legacy-Pb HI	0.434 (0.264–0.523)	0.273 (0.159–0.334)	0.156 (0.133–0.181); 37.1% (34.4–39.7)
Total-As cancer screen	1.49 × 10^−4^ (8.99 × 10^−5^–1.94 × 10^−4^)	1.01 × 10^−4^ (5.82 × 10^−5^–1.29 × 10^−4^)	5.49 × 10^−5^ (4.54 × 10^−5^–6.50 × 10^−5^); 35.9% (32.4–39.2)
Primary HI > 1, *n* (%)	0/34 (0%)	0/34 (0%)	Not applicable
Total-As screen > 10^−4^, *n* (%)	22/34 (64.7%)	17/34 (50.0%)	5 fewer; 14.7 percentage-point decrease

Continuous strategy outcomes are median (IQR). The reduction column reports the mean within-sample absolute reduction followed by the mean within-sample relative reduction, each with a sample-level bootstrap 95% CI. Threshold rows are counts (percentages).

**Table 4:** External Turkish evidence and limits of comparability.

Study	Samples/Protocol	Common Information	Use in This Study
Hamitoğlu et al., 2026 [14]	8 brands; 6 g/200 mL; 5 min	As, Cd, Pb, Cu, Zn; HI 0.17–0.25	Quantitative study-level context; no pooling
Başaran et al., 2023 [11]	10 brands; light/normal/over-brewed	Brew strength and health-risk assessment	Qualitative sensitivity context
Alkayın and Yıldız 2022 [12]	54 black teas; 1 g/40 mL; 20 min	Al infusion; Pb, Cr, Co, Ni, Cd in leaves	Regional matrix context
Demirdöğen and Kutlu 2023 [13]	Turkish teas; multiple brewing conditions	Al, Mn, Fe, Cu, Zn, Ni	Element and protocol context
Polat et al., 2022 [4]	Traditional double-teapot; infusion-time experiment	Cu, Zn, Al, Ca, Mn; chemical-quality and sensory outcomes	Brewing-protocol evidence; no risk validation
Cho et al., 2026 [5]	Factorial double-teapot experiment; 15–20 min; diluted serving	Vessel, temperature, addition order, dilution; sensory and chemical quality	Brewing-protocol evidence; no toxic-element risk validation

Exposure/risk studies and brewing-protocol experiments are shown separately. They differ in tea form, brewing ratio, duration, measured outcomes, analytical platform, body weight, and exposure equations; the brewing studies are not external validation datasets.

## Data Availability

Source measurements are publicly available from Mendeley Data, Version 1 (DOI: 10.17632/4md98yy6c4.1), under CC BY 4.0. The data supporting this study’s findings are available in the article and its Supplementary Materials, including all derived datasets and analysis scripts generated during this study.

## Supplementary Materials

The following supporting information can be downloaded at: https://www.mdpi.com/article/10.3390/foods15203613/s1, Table S1: Sequential-infusion profiles in black tea; Table S2: First-infusion share of cumulative three-infusion yield; Table S3: Zero measurements and element-specific effective sample sizes; Table S4: First-infusion share sensitivity restricted to complete-positive observations; Table S5: Estimated reduction in the brewed yield of Cu, Se, and Zn after discarding the first infusion; Table S6: Primary and legacy-Pb sensitivity risk results; Table S7: Sensitivity to assumed inorganic-arsenic fraction; Table S8: Intake and body-weight sensitivity scenarios; Table S9: Equal-80-mL sensitivity comparison; Table S10: Primary and all-tea sensitivity outcomes; Table S11: Country heterogeneity of paired HI reduction in black tea; Table S12: Influence diagnostics for mean relative reductions in black tea (*n* = 34); Table S13: Comparison with the associated source publication and the closest independent successive-infusion study; Figure S1: Sample-level distributions of each element’s share across the three sequential infusions. Red horizontal bars indicate medians; each blue point is one tea sample with a defined three-infusion total; Supplementary Code S1: Python (version 3.12) scripts, fixed-seed instructions, dependency information, source and derived CSV files, and reproduction instructions.

## Abbreviations

ADD	Average daily dose
Ag	Silver
AI	Artificial intelligence
Al	Aluminium
As	Arsenic
Ba	Barium
BH	Benjamini–Hochberg
BW	Body weight
Ca	Calcium
CC BY	Creative Commons Attribution
Cd	Cadmium
CI	Confidence interval
Co	Cobalt
Cr	Chromium
Cr(VI)	Hexavalent chromium
CSF	Cancer slope factor
Cu	Copper
DOI	Digital object identifier
EFSA	European Food Safety Authority
FAO	Food and Agriculture Organization of the United Nations
Fe	Iron
HI	Hazard index
HQ	Hazard quotient
ICP-OES	Inductively coupled plasma optical emission spectrometry
IQR	Interquartile range
IR	Ingestion rate
JECFA	Joint FAO/WHO Expert Committee on Food Additives
Mn	Manganese
Ni	Nickel
Pb	Lead
PTWI	Provisional tolerable weekly intake
RDA	Recommended dietary allowance
RfD	Reference dose
Se	Selenium
WHO	World Health Organization
Zn	Zinc

## References

[1] Na Nagara V., Sarkar D., Luo Q., Biswas J.K., Datta R. (2022). Health risk assessment of exposure to trace elements from drinking black and green tea marketed in three countries. Biol. Trace Elem. Res..

[2] Hu C., Zhang X., Zhan N., Liu Y. (2023). Current status and health risk assessment of heavy metals contamination in tea across China. Toxics.

[3] Scutarasu E.C., Trinca L.C. (2023). Heavy metals in foods and beverages: Global situation, health risks and reduction methods. Foods.

[4] Polat A., Kalcıoğlu Z., Müezzinoğlu N. (2022). Effect of infusion time on black tea quality, mineral content and sensory properties prepared using traditional Turkish infusion method. Int. J. Gastron. Food Sci..

[5] Cho J.M., Yavuz Abanoz Y., Boyraz A., Demir A., Çavuş D., Er Çapoğlu H., Kopuz Kıyak A., Cam Ş., Mehdizadehtapeh L., Başaran B. (2026). Effects of brewing conditions on the sensory and chemical properties of Turkish black tea and their interrelationships. Sci. Rep..

[6] Zhang J., Yang R., Chen R., Peng Y., Wen X., Gao L. (2018). Accumulation of heavy metals in tea leaves and potential health risk assessment: A case study from Puan County, Guizhou Province, China. Int. J. Environ. Res. Public Health.

[7] International Agency for Research on Cancer (2012). Arsenic, Metals, Fibres and Dusts. IARC Monographs on the Evaluation of Carcinogenic Risks to Humans, Volume 100C.

[8] World Health Organization (2022). Arsenic. Fact Sheet. https://www.who.int/news-room/fact-sheets/detail/arsenic.

[9] European Food Safety Authority (EFSA) Panel on Contaminants in the Food Chain (2024). Update of the risk assessment of inorganic arsenic in food. EFSA J..

[10] Xiao J., Zhang D., Shen J., Gong W., Wu X., Wang F., Chen Y., Li X., Zheng Q., Luo D. (2023). Trace elements in successive tea infusions made via a brewing method widespread in China: Implications for human exposure. J. Food Compos. Anal..

[11] Başaran B., Yavuz Abanoz Y., Şenol N.D., Yılmaz Oral Z.F., Öztürk K., Kaban G. (2023). The levels of heavy metal, acrylamide, nitrate, nitrite, N-nitrosamine compounds in brewed black tea and health risk assessment: Türkiye. J. Food Compos. Anal..

[12] Alkayın M., Yıldız N. (2022). Determination of aluminum accumulation and heavy metal content of some tea produced in Eastern Black Sea Region [In Turkish: Doğu Karadeniz Yöresinde Üretilen Bazı Çayların Alüminyum Akümülasyonu ve Ağır Metal İçeriklerinin Belirlenmesi]. Ordu Univ. J. Sci. Technol..

[13] Demirdöğen R.E., Kutlu E. (2023). Investigation of heavy metal content of Turkish teas and tea infusions. Karatekin Univ. J. Sci..

[14] Hamitoğlu M., Şahin M., Esen G., Helvacıoğlu S., Aydın A. (2026). Assessment of the potential health risks associated with the toxic and essential elements content in tea samples marketed in Türkiye. Turk. J. Pharm. Sci..

[15] Na Nagara V., Sarkar D., Luo Q., Datta R. (2021). Heavy Metal Assessment of Tea Leaves and Infusions. Mendeley Data, Version 1. https://data.mendeley.com/datasets/4md98yy6c4/1.

[16] (1989). Risk Assessment Guidance for Superfund, Volume I: Human Health Evaluation Manual (Part A).

[17] U.S. Environmental Protection Agency Integrated Risk Information System (IRIS). https://www.epa.gov/iris.

[18] Joint FAO/WHO Expert Committee on Food Additives (2011). Evaluation of Certain Food Additives and Contaminants: Seventy-Third Report.

[19] European Food Safety Authority Panel on Contaminants in the Food Chain (2010). Scientific opinion on lead in food. EFSA J..

[20] Yuan C., Gao E., He B., Jiang G. (2007). Arsenic species and leaching characters in tea (*Camellia sinensis*). Food Chem. Toxicol..

[21] Mania M., Szynal T., Rebeniak M., Wojciechowska-Mazurek M., Starska K., Strzelecka A. (2014). Human exposure assessment to different arsenic species in tea. Rocz. Panstw. Zakl. Hig..

[22] Benjamini Y., Hochberg Y. (1995). Controlling the false discovery rate: A practical and powerful approach to multiple testing. J. R. Stat. Soc. Ser. B.

[23] Efron B., Tibshirani R.J. (1993). An Introduction to the Bootstrap.

[24] National Institutes of Health, Office of Dietary Supplements Copper: Fact Sheet for Health Professionals. https://ods.od.nih.gov/factsheets/Copper-HealthProfessional/.

[25] National Institutes of Health, Office of Dietary Supplements Selenium: Fact Sheet for Health Professionals. https://ods.od.nih.gov/factsheets/Selenium-HealthProfessional/.

[26] National Institutes of Health, Office of Dietary Supplements Zinc: Fact Sheet for Health Professionals. https://ods.od.nih.gov/factsheets/Zinc-HealthProfessional/.

[27] Institute of Medicine (2001). Dietary Reference Intakes for Vitamin A, Vitamin K, Arsenic, Boron, Chromium, Copper, Iodine, Iron, Manganese, Molybdenum, Nickel, Silicon, Vanadium, and Zinc.

[28] Institute of Medicine (2000). Dietary Reference Intakes for Vitamin C, Vitamin E, Selenium, and Carotenoids.

[29] Hu S., Jin L., Huang L., Liu C., Liang M., Liu H. (2026). Tea rinsing effectively mitigates probabilistic health risks from heavy metal exposure. Biol. Trace Elem. Res..

[30] Esfarjani F., Rouzbahani M., Behbahan S.E.B., Shahbazpour N., Moslemi M., Abedi A.S. (2024). Health risk assessment of heavy metals in black tea infusion by Monte Carlo simulation. Food Sci. Nutr..

[31] Ahmed M., Ahmad M., Khan M.A., Sohail A., Sanaullah M., Ahmad W., Iqbal D.N., Khalid K., Wani T.A., Zargar S. (2024). Assessment of carcinogenic and non-carcinogenic risk of exposure to potentially toxic elements in tea infusions: Determination by ICP-OES and multivariate statistical data analysis. J. Trace Elem. Med. Biol..

[32] European Food Safety Authority (EFSA) Scientific Committee (2024). Guidance on risk–benefit assessment of foods. EFSA J..

[33] Zhang X., Yusuf A., Xu M., Isah M.B. (2026). Three successive infusions: Scientific insights into a traditional green tea drinking custom. J. Food Sci..

[34] Długaszek M., Mierczyk J. (2024). Elemental composition of green tea infusions depending on the method of their brewing. Eur. Food Res. Technol..

[35] Wu S., Peng Z., Ou Y., Mhungu F., Wang Y., Zhang Y., Liu L., Lei J., Huang L., Peng R. (2025). Investigation of the impact of brewing parameters on toxic element and rare earth element contamination in oolong tea. Front. Nutr..

