# Supplementary material for: Sequential Infusions of Black Tea: Multi-Element Exposure Trade-Offs of Discarding the First Brew
Source: Foods. 2026 Oct 11;15(20):3613. doi: 10.3390/foods15203613 (PMC13704546; doi:10.3390/foods15203613)
Supplement: Supplementary file 1 [file foods-15-03613-s001.zip › Supplementary_Materials_Tables_S1-S13_and_Figure_S1_09102026.pdf]

Supplementary Materials

Sequential Infusions of Black Tea: Multi-Element Exposure Trade-Offs of Discarding the First Brew

Rüveyda Esra Özkalaycı and Fatih Özkalaycı

Contents: Supplementary Methods; Tables S1-S13; Figure S1. Supplementary Code S1 is provided as a separate ZIP archive.

Data source: Mendeley Data, Version 1; DOI: 10.17632/4md98yy6c4.1; CC BY 4.0.

Primary population: 34 black teas (China n = 10; India n = 15; United States n = 9), each with three linked sequential infusions.

Supplementary Methods

The source experiment used 1.5 g dry tea and 0.04 L per infusion. Measured concentrations (micrograms/L) were converted to dry-tea-equivalent content (mg/kg) as concentration x volume/dry mass. Cumulative content summed linked infusions. The discard-first strategy retained the second and third infusions. The source publication reported duplicate sample preparation and ICP-OES measurement; however, replicate-level values and sample-specific detection limits were not separately available in the deposited worksheet.

The primary scenario used 10 g dry tea/day and 65 kg body weight. Sensitivity scenarios crossed 5, 10, and 15 g/day with 65, 70, 75, 80, and 85 kg, yielding 15 intake-body-weight combinations for each strategy. Primary HI excluded Pb; a legacy Pb-HQ HI was retained only for sensitivity. Equal-volume analysis compared the observed second-plus-third 80 mL with two-thirds of the pooled 120-mL all-infusion mass.

Infusion-order effects used 20,000 within-sample Friedman permutations, and first-infusion-share confidence intervals used 10,000 sample-level bootstrap resamples. These analyses used the original fixed seed 20260830 and preserved their interleaved execution order. Strategy effects are reported as absolute and relative paired changes with 20,000 sample-level bootstrap intervals using seed 20260930; nested-strategy p values were removed. Benjamini-Hochberg control was applied across the nine infusion-order tests. Arsenic sensitivity assumed 10%, 20%, 29%, 50%, 75%, 88%, or 100% inorganic As; total-As-based As HQ was reported separately. Mean strategy effects were additionally subjected to leave-one-sample-out and leave-one-country-out influence analyses.

Zeros were retained as reported. First-infusion shares were calculated only when the three-infusion total was positive. Zero counts, effective n, and complete-positive sensitivity estimates are reported below. The absence of sample-specific detection limits prevented substitution-based censored-data analysis.

Supplementary Code S1 contains five analysis scripts organized by function—core analysis, infusion-order analysis, toxicological sensitivity, essential-element yield, and robustness—together with all derived CSV tables, fixed random seeds, dependency versions, and reproduction instructions. The source workbook is identified by DOI and is not silently replaced or reconstructed.

Tables S1-S13

Table S1. Sequential-infusion profiles in black tea.

Element	First median	Second median	Third median	Friedman χ^2	Kendall W	Permutation p (minimum attainable 5e-05)	BH q
Ag	0.403	0.34	0.158	23.353	0.343	5e-05	7.5e-05
As	8.69	8.5	6.38	10.294	0.151	0.0045	0.005785
Ba	27.4	26.2	11.6	53.118	0.781	5e-05	7.5e-05
Cd	0	0	0	5.903	0.087	0.05055	0.05687
Cr	6.26	6.64	4.08	44.133	0.649	5e-05	7.5e-05
Cu	80.6	65.3	28.6	54.765	0.805	5e-05	7.5e-05
Pb	2.7	2.66	2.4	1.824	0.027	0.4176	0.4176
Se	5.13	3.72	1.98	19.143	0.282	5e-05	7.5e-05
Zn	165	186	80.3	40.235	0.592	5e-05	7.5e-05

Table S2. First-infusion share of cumulative three-infusion yield.

Element	Interpretive class	Median share (%)	95% bootstrap CI	Mean share (%)
Ag	Other/possibly toxic	38.8	35.7–42.3	39.8
As	Other/possibly toxic	37.1	32.4–40.3	35.9
Ba	Other/possibly toxic	48.2	41.6–53.8	49.1
Cd	Other/possibly toxic	36.4	33.0–47.1	36.3
Cr	Other/possibly toxic	37.3	35.2–43.2	42.5
Cu	Essential	48.0	43.2–52.6	48.1
Pb	Other/possibly toxic	36.7	31.4–40.7	35.8
Se	Essential	45.6	39.0–52.2	43.8
Zn	Essential	37.8	32.4–53.4	43.1

Table S3. Zero measurements and element-specific effective sample sizes.

Element	Infusion	Zero n (%)	Share effective n	Complete-positive n
Ag	first	0 (0.0%)	34	34
Ag	second	0 (0.0%)	34	34
Ag	third	0 (0.0%)	34	34
As	first	0 (0.0%)	34	34
As	second	0 (0.0%)	34	34
As	third	0 (0.0%)	34	34
Ba	first	0 (0.0%)	34	34
Ba	second	0 (0.0%)	34	34
Ba	third	0 (0.0%)	34	34
Cd	first	20 (58.8%)	16	13
Cd	second	18 (52.9%)	16	13
Cd	third	20 (58.8%)	16	13
Cr	first	0 (0.0%)	34	31
Cr	second	2 (5.9%)	34	31
Cr	third	2 (5.9%)	34	31
Cu	first	0 (0.0%)	34	34
Cu	second	0 (0.0%)	34	34
Cu	third	0 (0.0%)	34	34
Pb	first	0 (0.0%)	34	33
Pb	second	1 (2.9%)	34	33
Pb	third	0 (0.0%)	34	33
Se	first	6 (17.6%)	28	27
Se	second	6 (17.6%)	28	27
Se	third	7 (20.6%)	28	27
Zn	first	0 (0.0%)	34	34
Zn	second	0 (0.0%)	34	34
Zn	third	0 (0.0%)	34	34

Table S4. First-infusion share sensitivity restricted to complete-positive observations.

Element	Primary n	Primary median share (%)	Complete-positive n	Complete-positive median share (%)
Ag	34	38.8	34	38.8
As	34	37.1	34	37.1
Ba	34	48.2	34	48.2
Cd	16	36.4	13	38.6
Cr	34	37.3	31	36.5
Cu	34	48.0	34	48.0
Pb	34	36.7	33	36.8
Se	28	45.6	27	44.6
Zn	34	37.8	34	37.8

Table S5. Estimated reduction in the brewed yield of Cu, Se, and Zn after discarding the first infusion.

Element	Estimated median reduction in brewed yield, micrograms/day (IQR)	Adult RDA comparison
Cu	21.50 (16.92-27.72)	2.4% of 900 micrograms
Se	1.37 (0.59-2.19)	2.5% of 55 micrograms
Zn	43.93 (31.28-75.95)	0.5% of 8 mg (women); 0.4% of 11 mg (men)

Table S6. Primary and legacy-Pb sensitivity risk results.

Outcome	All median (IQR)	Discard median (IQR)	Mean absolute reduction (95% CI)	Mean relative reduction (95% CI)
HI excluding Pb (primary)	0.419 (0.258-0.497)	0.26 (0.157-0.326)	0.152 (0.128-0.177)	37.1% (34.4-39.8)
HI including legacy Pb HQ (sensitivity)	0.434 (0.264-0.523)	0.273 (0.159-0.334)	0.156 (0.133-0.181)	37.1% (34.4-39.7)
Total-As screening cancer risk	0.000149 (8.99e-05-0.000194)	0.000101 (5.82e-05-0.000129)	5.49e-05 (4.54e-05-6.5e-05)	35.9% (32.4-39.2)

Table S7. Sensitivity to assumed inorganic-arsenic fraction.

Inorganic As fraction	Strategy	Median (IQR)	>1e-4
10%	All three infusions	1.49e-05 (8.99e-06-1.94e-05)	0/34 (0.0%)
10%	Discard first	1.01e-05 (5.82e-06-1.29e-05)	0/34 (0.0%)
20%	All three infusions	2.97e-05 (1.80e-05-3.87e-05)	0/34 (0.0%)
20%	Discard first	2.02e-05 (1.16e-05-2.59e-05)	0/34 (0.0%)
29%	All three infusions	4.31e-05 (2.61e-05-5.61e-05)	0/34 (0.0%)
29%	Discard first	2.93e-05 (1.69e-05-3.75e-05)	0/34 (0.0%)
50%	All three infusions	7.44e-05 (4.50e-05-9.68e-05)	8/34 (23.5%)
50%	Discard first	5.05e-05 (2.91e-05-6.47e-05)	0/34 (0.0%)
75%	All three infusions	1.12e-04 (6.74e-05-1.45e-04)	19/34 (55.9%)
75%	Discard first	7.57e-05 (4.36e-05-9.71e-05)	7/34 (20.6%)
88%	All three infusions	1.31e-04 (7.91e-05-1.70e-04)	21/34 (61.8%)
88%	Discard first	8.88e-05 (5.12e-05-1.14e-04)	13/34 (38.2%)
100%	All three infusions	1.49e-04 (8.99e-05-1.94e-04)	22/34 (64.7%)
100%	Discard first	1.01e-04 (5.82e-05-1.29e-04)	17/34 (50.0%)

Table S8. Intake and body-weight sensitivity scenarios.

Tea intake (g/day)	Body weight (kg)	Strategy	HI median (IQR)	HI > 1	As > 1e-4
5	65	All three infusions	0.210 (0.129-0.249)	0/34	7.44e-05 (4.50e-05-9.68e-05); 8/34 > 1e-4
5	65	Discard first	0.130 (0.078-0.163)	0/34	5.05e-05 (2.91e-05-6.47e-05); 0/34 > 1e-4
5	70	All three infusions	0.195 (0.120-0.231)	0/34	6.90e-05 (4.18e-05-8.98e-05); 6/34 > 1e-4
5	70	Discard first	0.121 (0.073-0.151)	0/34	4.69e-05 (2.70e-05-6.01e-05); 0/34 > 1e-4
5	75	All three infusions	0.182 (0.112-0.215)	0/34	6.44e-05 (3.90e-05-8.39e-05); 5/34 > 1e-4
5	75	Discard first	0.113 (0.068-0.141)	0/34	4.37e-05 (2.52e-05-5.61e-05); 0/34 > 1e-4
5	80	All three infusions	0.170 (0.105-0.202)	0/34	6.04e-05 (3.65e-05-7.86e-05); 2/34 > 1e-4
5	80	Discard first	0.106 (0.064-0.133)	0/34	4.10e-05 (2.36e-05-5.26e-05); 0/34 > 1e-4
5	85	All three infusions	0.160 (0.099-0.190)	0/34	5.69e-05 (3.44e-05-7.40e-05); 1/34 > 1e-4
5	85	Discard first	0.099 (0.060-0.125)	0/34	3.86e-05 (2.22e-05-4.95e-05); 0/34 > 1e-4
10	65	All three infusions	0.419 (0.258-0.497)	0/34	1.49e-04 (8.99e-05-1.94e-04); 22/34 > 1e-4
10	65	Discard first	0.260 (0.157-0.326)	0/34	1.01e-04 (5.82e-05-1.29e-04); 17/34 > 1e-4
10	70	All three infusions	0.389 (0.240-0.462)	0/34	1.38e-04 (8.35e-05-1.80e-04); 22/34 > 1e-4
10	70	Discard first	0.241 (0.146-0.303)	0/34	9.37e-05 (5.40e-05-1.20e-04); 14/34 > 1e-4
10	75	All three infusions	0.363 (0.224-0.431)	0/34	1.29e-04 (7.79e-05-1.68e-04); 21/34 > 1e-4
10	75	Discard first	0.225 (0.136-0.283)	0/34	8.75e-05 (5.04e-05-1.12e-04); 13/34 > 1e-4
10	80	All three infusions	0.341 (0.210-0.404)	0/34	1.21e-04 (7.31e-05-1.57e-04); 20/34 > 1e-4
10	80	Discard first	0.211 (0.127-0.265)	0/34	8.20e-05 (4.73e-05-1.05e-04); 12/34 > 1e-4
10	85	All three infusions	0.321 (0.198-0.380)	0/34	1.14e-04 (6.88e-05-1.48e-04); 19/34 > 1e-4
10	85	Discard first	0.199 (0.120-0.249)	0/34	7.72e-05 (4.45e-05-9.90e-05); 8/34 > 1e-4
15	65	All three infusions	0.629 (0.388-0.746)	1/34	2.23e-04 (1.35e-04-2.90e-04); 32/34 > 1e-4
15	65	Discard first	0.390 (0.235-0.489)	0/34	1.51e-04 (8.72e-05-1.94e-04); 22/34 > 1e-4
15	70	All three infusions	0.584 (0.360-0.692)	1/34	2.07e-04 (1.25e-04-2.70e-04); 30/34 > 1e-4
15	70	Discard first	0.362 (0.218-0.454)	0/34	1.41e-04 (8.10e-05-1.80e-04); 21/34 > 1e-4
15	75	All three infusions	0.545 (0.336-0.646)	0/34	1.93e-04 (1.17e-04-2.52e-04); 30/34 > 1e-4
15	75	Discard first	0.338 (0.204-0.424)	0/34	1.31e-04 (7.56e-05-1.68e-04); 19/34 > 1e-4
15	80	All three infusions	0.511 (0.315-0.606)	0/34	1.81e-04 (1.10e-04-2.36e-04); 29/34 > 1e-4
15	80	Discard first	0.317 (0.191-0.398)	0/34	1.23e-04 (7.09e-05-1.58e-04); 18/34 > 1e-4
15	85	All three infusions	0.481 (0.296-0.570)	0/34	1.71e-04 (1.03e-04-2.22e-04); 27/34 > 1e-4
15	85	Discard first	0.298 (0.180-0.374)	0/34	1.16e-04 (6.67e-05-1.48e-04); 18/34 > 1e-4

Table S9. Equal-80-mL sensitivity comparison.

Outcome	Mean reduction (95% CI)	Median reduction (IQR)
HI excluding Pb	5.7% (1.5 to 9.7)	6.4% (-1.9 to 13.7)
HI including legacy Pb HQ	5.7% (1.7 to 9.6)	6.5% (-1.3 to 13.6)
Total-As screening cancer risk	3.8% (-1.5 to 8.9)	5.6% (-6.5 to 12.6)

Table S10. Primary and all-tea sensitivity outcomes.

Population	Strategy	HI median (IQR)	As screening risk median (IQR)	HI > 1 (%)	As risk > 10^{-4} (%)
Black tea (n = 34)	All three infusions	0.434 (0.264–0.523)	1.49e-04 (8.99e-05–1.94e-04)	0.0	64.7
Black tea (n = 34)	Discard first	0.273 (0.159–0.334)	1.01e-04 (5.82e-05–1.29e-04)	0.0	50.0
All tea (n = 45)	All three infusions	0.447 (0.262–0.529)	1.64e-04 (9.14e-05–2.08e-04)	0.0	64.4
All tea (n = 45)	Discard first	0.257 (0.159–0.349)	9.76e-05 (5.48e-05–1.32e-04)	0.0	46.7

Table S11. Country heterogeneity of paired HI reduction in black tea.

Country	n	Mean reduction (%)	95% bootstrap CI	Median reduction (%)
China	10	37.4	33.8–41.0	36.8
India	15	37.5	33.8–41.1	37.6
USA	9	36.2	29.5–42.7	38.8

Table S12. Influence diagnostics for mean relative reductions in black tea (n = 34).

Outcome	Full mean (%)	Leave-one-sample-out range (%)	Maximum shift (percentage points)	Leave-one-country-out range (%)
HI reduction: all three vs discard first	37.1	36.6-37.7	0.55	36.8-37.4
Total-As screening reduction: all three vs discard first	35.9	35.2-36.6	0.74	35.0-36.2
HI reduction: equal 80 mL vs discard first	5.7	5.0-6.5	0.83	5.3-6.1
Total-As screening reduction: equal 80 mL vs discard first	3.8	2.8-4.9	1.11	2.5-4.3

Table S13. Comparison with the associated source publication and the closest independent successive-infusion study.

Dimension	Prior evidence	Present reanalysis
Question	Country, tea type, and infusion-process effects	Consumer strategy: all three versus discard first
Dose reconstruction	Workbook risk calculations by infusion	Cumulative linked-sample mass balance
Uncertainty	Source-study analyses	Paired bootstrap absolute and relative effects
Volume effect	Not separated	Equal-80-mL sensitivity
Pb	Historical PTWI proxy	Excluded from primary HI; separate exposure and legacy sensitivity
Arsenic	Total-As screening	10-100% inorganic-fraction sensitivity
Nutrition	Element concentrations	Estimated reduction in brewed yield of Cu, Se, and Zn relative to adult RDAs
Zeros	Workbook values	Zero counts, effective n, complete-positive sensitivity
Türkiye	Not a focus	Study-level context without pseudo-observations
Closest independent comparator	Xiao et al.: eight short infusions; successive-infusion element patterns and cumulative HI under a China-widespread protocol; different tea types, infusion durations, volumes, and element panel	Three linked 40-mL infusions in 34 black teas; discard-first consumer contrast; paired uncertainty, equal-volume decomposition, and toxicological sensitivity analyses

Figure S1

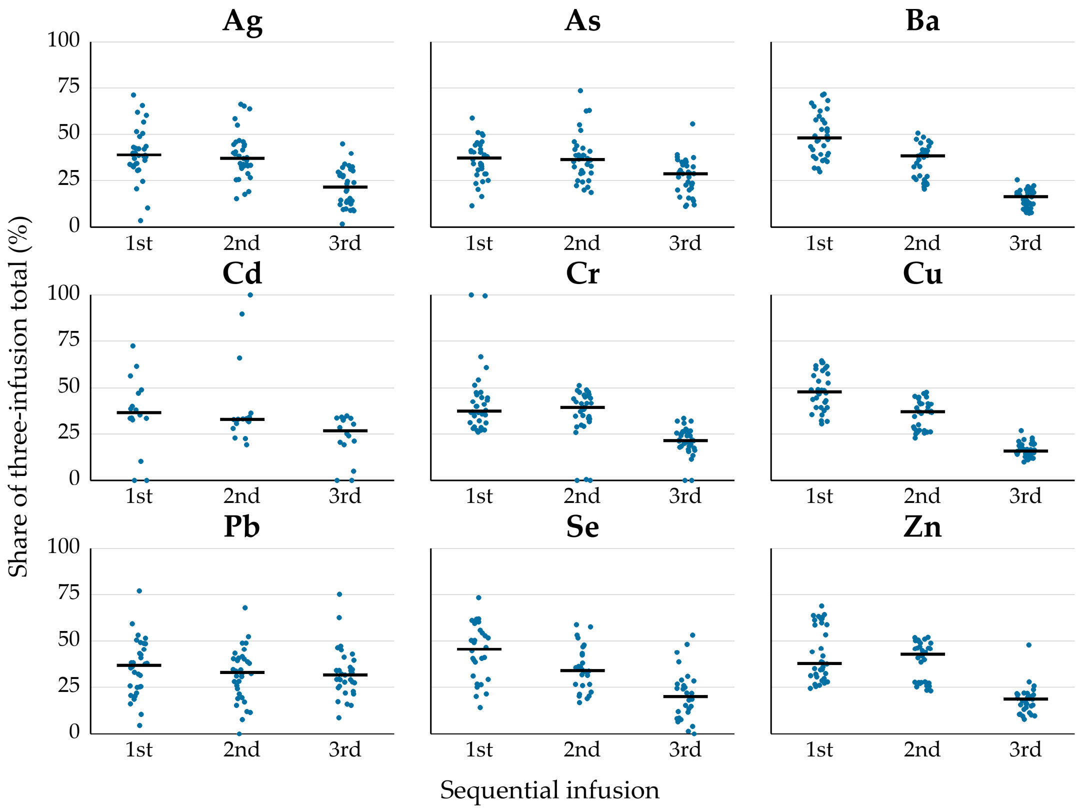

Figure S1. Sample-level distributions of each element's share across the three sequential infusions. Red horizontal bars indicate medians; each blue point is one tea sample with a defined three-infusion total.
